# Decompressive craniectomy combined with mild hypothermia in patients with large hemispheric infarction: a randomized controlled trial

**DOI:** 10.1186/s12883-021-02142-7

**Published:** 2021-03-12

**Authors:** Linlin Fan, Yingying Su, Yan Zhang, Hong Ye, Weibi Chen, Gang Liu

**Affiliations:** Department of Neurology, Xuanwu Hospital Capital Medical University, No. 45 Changchun Street, Xicheng District, Beijing, 100053 China

**Keywords:** Large hemispheric infarction, Decompressive craniectomy, Target temperature management, Randomized controlled trial, Neurological outcome

## Abstract

**Background:**

The effect of hypothermia on large hemispheric infarction (LHI) remains controversial. Our study aimed to explore the therapeutic outcomes of decompressive craniectomy (DC) combined with hypothermia on LHI.

**Methods:**

Patients were randomly divided into three groups: the DC group, the DC plus head surface cooling (DCSC) group and the DC plus endovascular hypothermia (DCEH) group. The DC group was maintained normothermia. The DCSC group received 24-h ice cap on the head for 7 days. While the DCEH group were given endovascular hypothermia (34 °C). Mortality and modified Rankin Scale (mRS) score at 6 months were evaluated.

**Results:**

Thirty-four patients were included in the study. Mortality of the DC, DCSC and DCEH groups at discharge were 22.2% (2/9), 0% (0/14) and 9.1% (1/11), respectively. However, it increased to 44.4% (4/9), 21.4% (3/14) and 45.5% (5/11) at 6 months, respectively (*p* = 0.367). Pneumonia (8 cases) was the leading cause of death after discharge. Twelve cases (35.3%) achieved good neurological outcome (mRS 0–3) at 6 months. The proportions of good neurological outcome in the DC, DCSC and DCEH groups were 22.2% (2/9 cases), 42.9% (6/14 cases) and 36.4% (4/11), respectively. The DCSC group seemed to have higher proportion of good outcomes, but there was no significant difference between groups (*p* = 0.598). Among survivors, endovascular hypothermia had a higher proportion of good outcome (DC group, 2/5 cases, 40.0%; DCSC group, 6/11 cases, 54.5%; DCEH group, 4/6 cases, 66.7%; *p* = 0.696). The incidence of complications in the DCEH group was higher than those of the DC and DCSC groups (18.9%, 12.0%, and 12.1%, respectively; *p* = 0.025).

**Conclusions:**

There is still no evidence to confirm that hypothermia further reduces long-term mortality and improves neurological outcomes in LHI patients with DC. However, there is a trend to benefit survivors from hypothermia. A local cooling method may be a better option for DC patients, which has little impact on systematic complications.

**Trial registration:**

Decompressive Hemicraniectomy Combined Hypothermia in Malignant Middle Cerebral Artery Infarct, ChiCTR-TRC-12002698. Registered 11 Oct 2012- Retrospectively registered, URL: http://www.chictr.org.cn/showproj.aspx?proj=6854.

## Background

Large hemispheric infarction (LHI) is the most malignant type of supratentorial ischemic stroke. The mortality rate in these patients is as high as 53 to 78%, even after the strongest available medical treatments [1–5]. Although randomized controlled trials (RCTs) have demonstrated that decompressive craniectomy (DC) can reduce mortality to 17 to 36%, the neurological outcomes in survivors, of whom 33.3 to 70.0% have an mRS of 4–5 [2–8], are not ideal.

Preclinical trials have demonstrated that mild hypothermia provides neuroprotective effect and reduces intracranial pressure (ICP). It also effectively prevents disruptions to the blood-brain barrier; reduces cerebral glucose metabolism, oxygen consumption, the accumulation of excitotoxic neurotransmitters, intracellular acidosis, intracellular calcium influx and oxygen-free radical production; alters the expression of “cold shock proteins”; reduces brain edema; minimizes the risk of thrombosis; and decreases the risk of epileptic activity [9–14]. Our RCT also showed that in LHI non-DC patients, better neurological outcomes were achieved in the surviving patients in the hypothermia group than in the control group (7/8, 87.5% versus 4/10, 40.0%, *p* = 0.066; OR = 10.5, 95% CI 0.9–121.4) [8]. These results demonstrate that mild hypothermia may improve neurological outcomes in survivors. Thus, we proposed that DC combined with mild hypothermia could improve both mortality and neurological outcomes in LHI patients. Therefore, we conducted this RCT to investigate the effect of DC combined with hypothermia treatment in LHI.

## Methods

### Patient population

From July 2010 to June 2016, patients with acute ischemic stroke in Department of Neurology, Xuanwu Hospital Capital Medical University were screened. We included 18 to 80 years old patients who involved ≥two-thirds of the middle cerebral artery (MCA) territory on cranial computed tomography or magnetic resonance imaging within 48 h after symptom onset. Meanwhile, the score of National Institutes of Health Stroke Scale (NIHSS) item 1a which reflected consciousness needed to be ≥1. DC was operated within 48 h of onset.

Exclusion criteria included large volume hemorrhage transformation, malignant herniation, severe coagulopathy, severe infection, and abnormal structure of inferior vena cava, etc., which could be referred to our previously published study [8].

### Randomization

This was a prospective, single-center RCT, including 3 groups: DC group, DC plus head surface cooling group (DCSC group) and DC plus endovascular hypothermia group (DCEH group). The random number was sealed in an envelope before the initiation of the study and was opened by an separate investigator.

### Standard medical treatment

Patients were admitted into the neurointensive care unit (NCU). Vital signs were monitored, head was elevated at 30° and 20% mannitol (125 mL every 4 h) was given to decrease ICP. Oxygenation, blood pressure, glucose were sustained at appropriate level. Early enteral nutrition was given. Pneumonia and deep venous thrombosis were monitored and well treated. The details of standard medical treatment could be referred to our previously published study [8].

### Decompressive craniectomy

All included patients received DC first as soon as possible. DC consisted of a large hemicraniectomy and a duraplasty. The bone flap with a diameter of at least 12 cm, which included temporal, frontal, parietal, and some occipital bones, was removed. The dura was opened, and a dural patch made of a dura substitute was placed into the incision and secured. Resection of infarcted brain tissue was forbidden.

### Temperature management

Patients allocated to the DCSC group were sustained using ice caps that were placed around the head. The ice cap was worn 24 h a day for 7 days. The systematic temperature was sustained normothermia.

Patients randomized to the DCEH group were treated with endovascular hypothermia. Hypothermia was initiated as soon as possible after DC. The target bladder temperature was 34 °C with maximal cooling rate. Hypothermia was maintained for 24 to 72 h according to the physician’s discretion. The rewarming was controlled 0.5 °C every 12 h until 36 °C was reached. If ICP rebounded during rewarming period, suspending the rewarming process until the patient regained stable.

The temperature of the patients in the DC group was sustained between 36.5 and 37.5 °C to maintain normothermia.

### Data collection

The baseline variables included age, sex, comorbidities (including hypertension, coronary heart disease, atrial fibrillation, hyperlipidemia, valvular dysfunction, diabetes mellitus and stroke), affected hemisphere, infarcted area, GCS score, NIHSS score and Acute Physiology And Chronic Health Evaluation II score, transtentorial herniation, the administration of thrombolysis or antiplatelet or anticoagulants or defibrinogen and the time interval from symptom onset to DC procedure.

We run blood routine tests, coagulation tests, blood biochemical tests and arterial blood gas analysis every 12 h in the DCEH group and every other day in the DC and DCSC group. The details of tests could be referred to our previously published study [8]. Chest radiography, electrocardiogram and deep venous ultrasound were performed every 3 days in all patients.

We also observed severe complications, including hemorrhagic transformation, recurrent infarction, transtentorial herniation post-DC, intracranial hemorrhage or infection post-DC, severe arrhythmia resulting hemodynamic disorder, heart failure (New York Heart Association class IV), hypotension (systolic blood pressure < 90 mmHg), pulmonary embolism, gastrointestinal bleeding requiring a blood transfusion (hemoglobin < 7 g/L), gastric retention (gastric residual > 250 mL), refractory hiccup, acute pancreatitis, acute liver injury, acute kidney injury, platelet < 50 × 10^9^/L, disseminated intravascular coagulation, infection (e.g., catheter-related infection, bacteremia, sepsis, pneumonia and urinary infection), severe electrolyte disorder (blood sodium > 160 or < 125 mmol/L, blood potassium > 6.5 or < 2.5 mmoL/L), stress hyperglycemia (> 11.1 mmol/L), hypoalbuminemia (< 30 g/L), and lower extremity deep vein thrombosis.

### Outcome measurement

All-cause mortality and mRS score at 6 months were investigated blindly by an separate investigator. Good neurological outcome was defined as a mRS 0–3. Complications were recorded as a secondary outcome.

### Statistical analysis

No similar results were found before the study was designed. Based on data from a previous study of hypothermia on stroke, assuming that the group difference of mean mRS was 1 and the standard deviation was 2 (α = 0.05; β = 0.10), the calculated sample size was 252. Because of the slow recruitment, the study was terminated early after a period of 6 years and we are planning a multicenter RCT in China.

Statistic Package for Social Science (SPSS) 25.0 (SPSS Inc., Chicago, Illinois, USA) was used for statistical analyses. The mean ± SD, median (range) or counts (proportions) were adopted, as appropriate. The two-tailed Kruskal-Wallis H test for continuous covariates and the Pearson’s chi-square test for categorical covariates were used when comparing between groups. All tests were two-tailed, and *p*-value < 0.05 was considered statistically significant.

## Results

### Patient characteristics

**Fig. 1:**
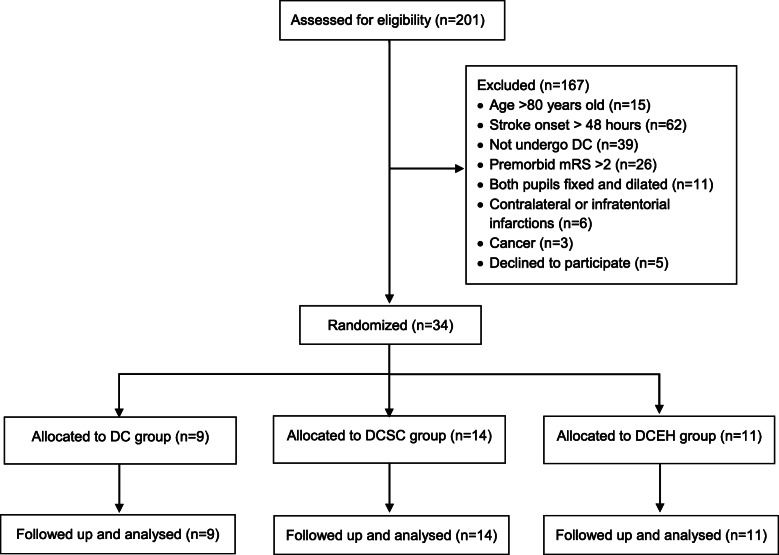
Flow chart of patient profile

Thirty-four patients were included in this trial. All included patients finished treatment and completed follow-up (Fig. 1). Eighteen patients (52.9%) were older than 60 years old. There were more male patients (28 cases, 82.4%) than female patients. The right hemisphere was affected in 20 patients (58.8%), and the total MCA or > MCA territory was involved in 24 patients (70.6%). Seven patients (20.6%) developed transtentorial herniation before inclusion.

There were 9 patients in the DC group, 14 patients in the DCSC group and 11 patients in the DCEH group. The patient baseline characteristics were listed in Table 1. There were no significant differences among the three groups.

**Table 1:** Patient Characteristics

Variables	DC (*n* = 9)	DCSC (*n* = 14)	DCEH (*n* = 11)	*P* value
Age, years, mean ± SD	63.22 ± 8.66	56.07 ± 11.23	58.18 ± 9.92	0.298
Male, n (%)	8 (88.9)	11 (78.6)	9 (81.8)	0.817
Comorbidity, n (%)				
Hypertension	4 (44.4)	4 (28.6)	3 (27.3)	0.663
Coronary heart disease	0	0	0	
Valvular dysfunction	1 (11.1)	2 (14.3)	2 (18.2)	0.905
Atrial fibrillation	4 (44.4)	5 (35.7)	3 (27.3)	0.726
Diabetes mellitus	2 (22.2)	1 (7.1)	1 (9.1)	0.519
Hyperlipidemia	0	1 (7.1)	0	0.479
Premorbid stroke	2 (22.2)	1 (7.1)	1 (9.1)	0.519
Left hemispheric affected, n (%)	4 (44.4)	5 (35.7)	5 (45.5)	0.863
Infarcted area, n (%)				
2/3 MCA	1 (11.1)	4 (28.6)	5 (45.5)	0.139
MCA	4 (44.4)	5 (35.7)	6 (54.5)	
> MCA	4 (44.4)	5 (35.7)	0	
GCS, mean ± SD	8.78 ± 2.73	10.71 ± 2.59	10.55 ± 2.54	0.198
NIHSS, mean ± SD	19.78 ± 2.64	17.64 ± 3.69	18.36 ± 3.01	0.337
APACHE II, mean ± SD	14.00 ± 4.18	10.43 ± 3.72	10.82 ± 4.73	0.163
Transtentorial herniation, n (%)	2 (22.2)	3 (21.4)	2 (18.2)	0.971
Prior T/A/A/D, n (%)	4 (44.4)	7 (50.0)	4 (36.4)	0.792
Interval time to DC^a^, hour, mean ± SD	23.28 ± 15.60	37.32 ± 12.21	30.05 ± 12.65	0.075

*MCA middle cerebral artery, GCS* glasgow coma scale, *NIHSS* National Institute of Health Stroke Scale, *APACHE II* acute physiology and chronic health evaluation II, *T/A/A/D* treatment with thrombolysis, antiplatelet, anticoagulant or defibrinogen, *DC* decompressive craniectomy

^a^Interval from symptom onset to DC: DC group vs. DCSC group, *P* = 0.025; DC group vs. DCEH group, *P* = 0.298; DCSC group vs. DCEH group, *P* = 0.159

### Mortality

The mortality rates of all patients at the time of discharge and after 6 months were 8.8% (3/34) and 35.3% (12/34), respectively. The mortality rates in the DC, DCSC and DCEH groups at the time of discharge were 22.2% (2/9), 0% (0/14) and 9.1% (1/11), respectively. There was no significant difference among the groups (*p* = 0.186). At 6 months, the mortality rates in the DC, DCSC and DCEH groups were 44.4% (4/9), 21.4% (3/14) and 45.5% (5/11), respectively (*p* = 0.367). The causes of death were described in the Complications section below.

### Neurological outcome

**Fig. 2:**
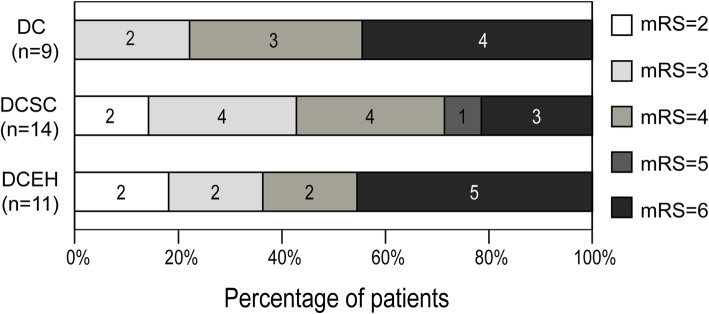
mRS at 6 months. At 6 months, the mortality rates in the DC, DCSC and DCEH groups were 44.4% (4/9), 21.4% (3/14) and 45.5% (5/11), respectively (*p* = 0.367). The rates of good neurological outcome (mRS 0–3) in the DC, DCSC and DCEH groups were 22.2% (2/9 cases), 42.9% (6/14 cases) and 36.4% (4/11), respectively. DCSC group seemed to have higher proportion of good outcome, but there was no statistical difference between groups (*p* = 0.598)

At 6 months, 12 cases (35.3%) had good neurological outcomes (mRS score of 0–3). The rates of good neurological outcome in the DC, DCSC and DCEH groups were 22.2% (2/9 cases), 42.9% (6/14 cases) and 36.4% (4/11), respectively. The DCSC group seemed to have a higher proportion of good outcome, but there was no significant difference between groups (*p* = 0.598). Among survivors, endovascular hypothermia had a higher proportion of good neurological outcome but also without significant difference (DC group, 2/5 cases, 40.0%; DCSC group, 6/11 cases, 54.5%; DCEH group, 4/6 cases, 66.7%; *p* = 0.696) (Fig. 2).

### Complications

The total cases that experienced complications in the DC, DCSC and DCEH groups were 28 (12.0%), 44 (12.1%), 54 (18.9%), respectively, and the differences between the groups were statistically significant (*p* = 0.025). The DCEH group experienced significantly more complications than the other two groups (Table 2). Twelve patients died of severe complications, and three of these patients died before discharge. In the DC group, herniation and gastrointestinal bleeding were the cause of death in 1 patient each. In the DCEH group, herniation was the cause of death in 1 patient. The other patients died after discharge from the NCU. Pneumonia was the cause of death in 2 patients of the DC group and 3 patients of the DCSC group. In the DCEH group, 3 patients died of pneumonia, and 1 patient died of intracranial infection associated with DC.

**Table 2:** Complications

Complications	DC (*n* = 9)	DCSC (*n* = 14)	DCEH (*n* = 11)	*P* value
Recurrent infarction, n (%)	0	0	2 (18.2)	
Transtentorial herniation post DC, n (%)	2 (22.2)	0	2 (18.2)	
Intracranial hemorrhage post DC, n (%)	0	3 (21.4)	2 (18.2)	
Intracranial infection post DC, n (%)	0	0	1 (9.1)	
Hypotension, n (%)	3 (33.3)	0	4 (36.4)	
Pneumonia, n (%)	8 (88.9)	10 (71.4)	11 (100.0)	0.126
Urinary infection, n (%)	0	1 (7.1)	0	
Bacteremia, n (%)	0	0	1 (9.1)	
Lower extremity deep venous thrombosis, n (%)	2 (22.2)	3 (21.4)	6 (54.5)	0.160
Gastrointestinal bleeding (Hb < 7 g/L), n (%)	2 (22.2)	0	0	
Gastric retention, n (%)	2 (22.2)	5 (35.7)	9 (81.8)	0.016
Refractory hiccup, n (%)	0	2 (14.3)	1 (9.1)	
Acute liver injury, n (%)	0	2 (14.3)	2 (18.2)	
Stress hyperglycemia, n (%)	1 (11.1)	5 (35.7)	3 (27.3)	0.425
Hypoalbuminemia, n (%)	8 (88.9)	13 (92.9)	10 (90.9)	0.947
All cases^a^, n (%)	28 (12.0)	44 (12.1)	54 (18.9)	0.025

There was no hemorrhagic transformation, severe arrhythmia resulting hemodynamic disorder, heart failure (NYHA level IV), pulmonary embolism, catheter-related infection, sepsis, acute pancreatitis, acute kidney injury, platelet < 50 × 10^9^/L, disseminated intravascular coagulation and severe electrolyte disorder

^a^Incidence of complications = all cases of complications / (26 × number of patients)

## Discussion

Our results showed that the mortality rates in the DC, DCSC and DCEH groups were 44.4% (4/9), 21.4% (3/14) and 45.5% (5/11), respectively, without significant difference. The proportions of good neurological outcome in the DC, DCSC and DCEH groups were 22.2% (2/9 cases), 42.9% (6/14 cases) and 36.4% (4/11), respectively, with no significant difference. The DCSC group seemed to have a higher proportion of good outcomes. The incidence of complications in the DCEH group was significantly higher than those of the DC and DCSC groups.

Similar to prior studies [2–8], our results also showed that DC decreased mortality in LHI patients (discharge, 8.8%; 6 months, 35.3%). Additionally, we found that patients in the DCSC and DCEH groups might have lower mortality rates than those in the DC group at the time of discharge. Transtentorial herniation (4 cases, 11.7%) was a severe complication that was associated with death in the acute phase. Therefore, mortality might be further decreased by combining DC with hypothermia during this period. However, we found that severe cerebral edema still did not resolve in some patients, even after they were treated with DC and hypothermia. Meta-analyses have found that hypertonic saline has a better effect than mannitol on decreasing the ICP [15, 16]. Additionally, studies using animals have shown that glibenclamide provided a better neuroprotective effect than DC or hypothermia treatment, and it prevented malignant cerebral infarction [17–19]. These methods might be an additional option. Our results also showed that lower mortality unfortunately did not extend to the 6-month follow-up. The meta-analysis of Chen et al. showed a similar result. Compared to DC alone, combining DC and hypothermia had a tendency to reduce short-term mortality (RR = 0.52, 95% CI 0.26 to 1.05, *p* = 0.07) but had no significant effects on long-term mortality (RR = 1.26, 95% CI 0.58 to 2.76, *p* = 0.56) or neurological outcomes (RR = 0.81, 95% CI 0.53 to 1.24, *p* = 0.34) [20]. We found that pneumonia was the primary cause of death (8 cases, 23.5%), and all of these deaths occurred after discharge. The DESTINY study had the same results: the mortality rate after 30 days was 12%, it increased to 18% after 6 months, and pneumonia was the main cause of death [2]. Once patients survive the acute phase, initiating subsequent long-term nursing care and strategies aimed at preventing pneumonia become particularly important, which might be the key to further lower mortality. Studies have shown that rehabilitation enhances airway protection and decreases the incidence of pneumonia [21, 22]. Hence, good long-term nursing care and rehabilitation are desired.

In preclinical trials, DC combined with brain hypothermia had significantly additional benefits for the neurologic outcome, infarct volume and degree of neuroinflammation than DC alone in the MCA infarction model [23]. In our results, among the 22 surviving patients, the hypothermia group had better neurological outcomes than the DC group (DC group, 2/5 cases, 40.0%; DCSC group, 6/11 cases, 54.5%; DCEH group, 4/6 cases, 66.7%). Although these results did not reach statistical significance, which might be due to the small sample size, they implied that hypothermia might improve neurological outcomes. This result was consistent with the findings of Thomas et al. In their study, patients in the hypothermia group tended to achieve better neurological outcomes than the normothermia group (NIHSS, 10 vs. 11; *p* = 0.08) [24]. The study, which compared endovascular hypothermia with normothermia in LHI patients with contraindications for DC, also found that hypothermia improved neurological outcomes in survivors (hypothermia, 87.5% vs. normothermia, 40.0%; *p* = 0.066) [8]. However, Neugebauer el al. conducted a multicenter RCT researching hypothermia in addition to DC in LHI patients and found that the mortality rates at day 14 were similar [hypothermia group, 19% (5/26 case); control group, 13% (3/24 cases); OR = 1.65, *p* = 0 .70], with no significant difference regarding functional outcome after 12 months of follow-up [25]. Furthermore, the hypothermia group suffered more complications. However, there is still controversy regarding the methodology [26], and the sample sizes of all the above studies are small. A well-designed large-sample multicenter RCT is needed.

Previous reports have shown that the complication rate of hypothermia is high [8]. Thus, we were concerned about the safety of combining DC with hypothermia treatment. Park et al. found that pneumonia was the most common adverse event in the hypothermia group (5/25 cases, 25%). Additionally, in some cases, hypothermia needed to be discontinued due to side effects (sepsis, hypotension or bradycardia) [27]. Our results showed that the rate of complications in the DCEH group was significantly higher than in the other two groups. Gastric retention was significantly higher due to anti-shivering drugs, which inhibits gastrointestinal motility. There also seemed to be more lower extremity deep venous thrombosis in the endovascular hypothermia group, which had an effect on coagulation function. Although the incidence of pneumonia was similar between groups, the pneumonia in the endovascular hypothermia group was more severe due to the use of anti-shivering drugs. Endovascular hypothermia indeed brought more complications. However, we surprisingly found that the DCSC method had only a small influence on the whole system, and patients suffered few severe complications. Thus, it might be the better option for LHI patients after DC.

There are shortcomings to our study. Because we included only patients who received DC, we might have introduced selection bias. Our study was an open RCT, and it was therefore impossible to blind physicians to the treatment assignments. As a result, it was possible that the physicians paid more attention to the patients in the hypothermia group. Additionally, the sample size was too small to make a conclusion. Moreover, the incidence of LHI was low, which made it difficult for a single center to collect a large number of samples. A multicenter RCT including a larger sample population will need to be conducted in the future to confirm our results.

## Conclusions

There is still no evidence to confirm that hypothermia further reduces long-term mortality and improves neurological outcomes in LHI patients with DC. However, there is a trend to benefit survivors from hypothermia. A local cooling method may be a better option for DC patients, which has little impact on systematic complications. A multicenter RCT is needed to confirm these results.

## Data Availability

The datasets used and analyzed during the current study are available from the corresponding author on reasonable request.

## Abbreviations

DC: Decompressive craniectomy
LHI: Large hemispheric infarction
DCSC: DC plus head surface cooling
DCEH: DC plus endovascular hypothermia
mRS: modified Rankin Scale
RCTs: Randomized controlled trials
ICP: Intracranial pressure
MCA: Middle cerebral artery
NIHSS: National institutes of health stroke scale
GCS: Glasgow coma scale
NCU: Neurointensive care unit
SPSS: Statistic package for social science
